# Clinical effects of immunization, bleeding, and albumin-based fluid therapy in horses used as immunoglobulin source to produce a polyspecific antivenom (Echitab-plus-ICP) towards venoms of African snakes

**DOI:** 10.1016/j.toxcx.2023.100158

**Published:** 2023-04-17

**Authors:** Rose Mary Huertas, Mauricio Arguedas, Juan Manuel Estrada, Edwin Moscoso, Deibid Umaña, Gabriela Solano, Mariángela Vargas, Álvaro Segura, Andrés Sánchez, María Herrera, Mauren Villalta, Cynthia Arroyo-Portilla, José María Gutiérrez, Guillermo León

**Affiliations:** a Laboratorio de Análisis Clínicos, Escuela de Medicina Veterinaria, Universidad Nacional de Costa Rica, Heredia, Costa Rica; b Instituto Clodomiro Picado, Facultad de Microbiología, Universidad de Costa Rica, San José, Costa Rica; c Hospital de Equinos, Especies Mayores y Terapias Regenerativas, Escuela de Medicina Veterinaria, Universidad Nacional de Costa Rica, Heredia, Costa Rica; d Departamento de Análisis Clínicos, Facultad de Microbiología, Universidad de Costa Rica, San José, Costa Rica

**Keywords:** Antivenom, Bleeding, Horse, Immunization, Snake venom, Hematological parameters

## Abstract

During the production of snake antivenoms, the animals used as immunoglobulin source are subjected to processes that could deteriorate their physical condition. Therefore, these conditions must be carefully designed and validated. In this work, the immunization and bleeding protocols applied to horses used to produce the African polyspecific antivenom EchiTAb-plus-ICP were evaluated regarding their effects on the horses' health. The study focused on horses that had been previously immunized with venoms and then received periodic booster venom injections for antivenom production. It was found that the periodic immunization with 5 mg of a mixture of venoms of *Bitis arietans*, *Echis ocellatus*, *Dendroaspis polylepis,* and *Naja nigricollis* did not induce systemic signs of envenomation, and only caused mild swelling at the injection site, which did not evolve to abscesses, fistulas, or fibrosis. Three consecutive days of bleeding, collecting 6–8 L of blood per day, and self-transfusing the red blood cells (RBC) in the second and third days, did not induce evident cardiorespiratory alterations. However, this procedure caused significant reductions in RBC, hematocrit, hemoglobin, and total plasma protein values. Seven weeks after bleeding, these parameters were recovered, and horses were ready for the next immunization/bleeding cycle. The intravenous administration of equine albumin, at a dose of 2 g/kg body weight, increased the apparent plasma volume and the albumin concentration. However, this procedure induced early adverse reactions and transient alterations of the serum levels of the enzyme gamma-glutamyl transferase (GGT), thus suggesting some degree of hepatic injury. It was concluded that immunization and bleeding as described in this work do not cause significant clinical alterations in the horse's health, except for a transient drop in some hematological parameters. The albumin-based fluid therapy used does not hasten the recovery after bleeding but instead induces adverse events in the animals.

## Highlights

• Periodic immunization with snake venoms and bleeding has the potential to produce damage in horses.
• Doses of 5 mg of a mixture of venoms of *B. arietans*, *E. ocellatus*, *D. polylepis*, and *N. nigricollis* were safe.
• Collection of 6–8 L of blood during three consecutive days, with self-transfusions in the second and third days was safe.
• A rest period of seven weeks was enough for the horses to recover and be ready for the next immunization/bleeding cycle.
• The albumin-based fluid therapy did not accelerate recovery after bleeding but induced adverse events.

## Graphical abstract

**Figure:**
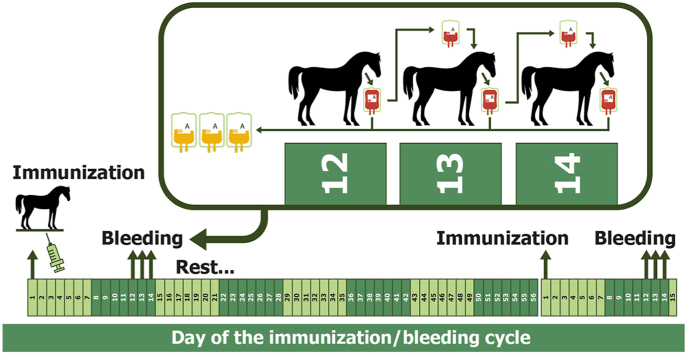

## 1 Introduction

Snake antivenoms are formulations of immunoglobulins able to bind toxins of snake venoms and neutralize their ability to induce tissue damage and other pathophysiological alterations (WHO, 2016). The typical stages of antivenom manufacture are: 1) Production and validation of snake venoms, 2) immunization of the animal source of immunoglobulins, 3) harvesting of hyperimmune plasma, 4) purification of immunoglobulins, 5) formulation and sterilization of the bulk, 6) filling and lyophilization, 7) labeling and packaging, and 8) releasing of the final product for human use and pharmacovigilance (León et al., 2018).

The immunization with snake venoms is designed to stimulate the immune system of the animals selected as immunoglobulin source (e.g., horses or sheep) to produce high amounts of neutralizing antibodies. Traditionally, this process is done by the repeated injection of small doses of venoms mixed with immunological adjuvants (e.g., emulsions or mineral salts) or simply dissolved in saline solution. The booster frequency and the amount of venom per injection is empirically established by each manufacturer (da Silva and Tambourgi, 2011; León et al., 2011), depending on the product profile.

During immunization, some adjuvants (e.g., Montanide or Freund's adjuvants) cause local tissue damage such as granulomas and abscesses, and general reactions such as arthritis and fever (Fukanoki et al., 2001; Jansen et al., 2007; Arguedas et al., 2022). Commonly, these abscesses fistulize, exposing the damaged tissue to secondary infections that hinder healing. For this reason, some manufacturers avoid the use of adjuvants when administering booster doses of venoms after the first immunization cycle. Additional local and systemic damage can be produced by the toxins released from the immunogenic implant and by the inflammatory response of the animal to venoms and adjuvants (Estrada et al., 1992; Angulo et al., 1997a; León et al., 1999; Waghmare et al., 2014).

To reduce the damage caused by venoms during immunization, some researchers have suggested the use of physicochemical methods to inactivate venom toxins. However, few producers have implemented this practice owing to the risk of affecting the antigenicity of the toxins and consequently reducing the eﬃcacy of the antivenoms towards native toxins (Theakston et al., 2003). A better alternative to reduce tissue damage during immunization while generating large amounts of neutralizing antibodies could be the low dose/low volume/multi-site protocol (Chotwiwatthanakun et al., 2011), but this must be demonstrated for each antivenom formulation.

After immunization, the harvest of hyperimmune plasma from healthy animals is performed by bleeding. At Instituto Clodomiro Picado the standard bleeding involves the collection of 6–8 L of blood during three consecutive days, with self-transfusion of red blood cells (RBC) resuspended in saline solution in the second and third days. Depending on the size and the hematological status of the animals, this process yields 8–12 L plasma per horse (Angulo et al., 1997b). Since hypoproteinemia, anemia, chronic dehydration, and eventually acute hemodynamic disturbances can occur because of this bleeding schedule, the safety of animals submitted to bleeding must be demonstrated (Angulo et al., 1997b).

The time it takes the horses to recover after the immunization and bleeding cycles determines the number of bleeding sessions that can be performed per year, affecting the animal productivity (i.e., kg hyperimmune plasma/horse/year). An interval of 8–10 weeks between bleedings is the normal recovery time for horses used in antivenom production. However, this lapse must be experimentally validated for each horse breed and immunization/bleeding procedures.

Since a significant component of the production cost of antivenoms is the production of hyperimmune plasma, the proper balance between animal welfare and its productivity is essential to reduce the production costs and, therefore, the sale price. In consequence, this will improve the accessibility of antivenoms to communities and health systems, while ensuring the well-being of animals. Therefore, developing strategies that speed up the recovery of horses and reduce the time between production cycles would benefit antivenom production.

The effects of immunization and bleeding on animal health have been studied in horses used to produce an antivenom towards the venoms of *Bothrops asper*, *Crotalus simus,* and *Lachesis stenophrys* (Estrada et al., 1992; Angulo et al., 1997a; 1997b). However, these results cannot be extrapolated to horses immunized with other venoms. Immunization of animals for antivenom production starts with an initial cycle of immunization in naïve horses. A previous study evaluated the impact of this initial immunization, using various adjuvants, in the health of horses that receive venoms of African snakes for the production of EchiTAb-plus-ICP antivenom (Arguedas et al., 2022). After the initial immunization scheme, horses are submitted to periodic booster injections of venom for continuous antivenom production. In the present work we have evaluated the effect of these booster immunizations in the health of horses used to produce the polyspecific antivenom EchiTAb-plus-ICP, which is distributed to several countries in sub-Saharan Africa. Moreover, we determined the effect of bleeding and of an albumin-based fluid therapy on the time required by these horses to recover and be ready for the next immunization/bleeding cycle.

## 2 Materials and methods

### 2.1 Ethics

This manuscript presents an experimental study performed following the standard procedures of scientific ethics, including those related to the use and care of animals. All procedures performed in this study meet the International Guiding Principles for Biomedical Research Involving Animals (CIOMS, 1985). All procedures involving animals (mice and horses) were approved by the Institutional Committee for the Care and Use of Laboratory Animals of Universidad de Costa Rica (approval code CICUA 202–2020).

### 2.2 Horses for experimentation

Thirty-eight creole horses (400–450 kg body weight) of both sexes, routinely used to produce EchiTAb-plus-ICP, were used to study the clinical effects induced during immunization and bleeding. Only six of these horses were used to study the effect of albumin-based fluid therapy in the recovery after bleeding. During experimentation, the horses were maintained in a grazing land at 1495 m above sea level, with access to water and pasture *ad libitum*, at a population density of 2 horses/Ha. The grazing technique was in paddocks with steep slopes, planted with Ray-grass forage. The diet was supplemented with pelleted feed enriched with proteins, vitamins, and minerals.

### 2.3 Immunization, bleeding, and albumin-based fluid therapy

#### 2.3.1 Immunization

The horses used in this study are part of the regular immunization schemes at Instituto Clodomiro Picado. Initially, new horses are subjected to immunization following the scheme described by Arguedas et al. (2022). After seven weeks at rest following immunization with venoms and bleeding, the horses were re-immunized by the subcutaneous injection of 5 mg of a mixture in equal parts of venoms of adult specimens of *Bitis arietans* (Latoxan, batch #322.061), *Echis ocellatus* (Latoxan, batch #200.171), *Dendroaspis polylepis* (Latoxan, batch #416.031), and *Naja nigricollis* (Latoxan, batch #616.031) (Gutiérrez et al., 2005). Lyophilized venoms were dissolved in 0.15 mol/L NaCl (saline solution) and sterilized by filtration immediately before subcutaneous injection in a single site in the back of horses. The injection volume was 2 mL per horse. No adjuvants are used in these booster immunizations.

#### 2.3.2 Bleeding

Twelve days after immunization, horses were submitted to three consecutive days of bleeding. On the first day, 6 L of blood was collected from the jugular vein of each horse by using a system of two PVC blood bags and citrate dextrose solution (ACD) (i.e., citric acid 0.093 mol/L, sodium citrate 0.197 mol/L and dextrose 0.6 mol/L) as an anticoagulant. Blood was stored at 2–8 °C overnight to allow red blood cells (RBC) to sediment. On the second day, plasma was separated from RBC after spontaneous sedimentation of cells. Plasma was preserved by the addition of 0.005% thimerosal, and stored at 2–8 °C. In turn, RBCs were re-suspended in 3 L of saline solution and warmed to 37 °C. Then, horses were submitted to a second collection of 6 L of blood followed by the self-transfusion of RBCs collected on the first day. On the third day, plasma collected on the second day was separated from RBCs, preserved, and stored; RBCs were re-suspended in saline solution and warmed to 37 °C. Then, horses were submitted to a third collection of 6 L of blood and the self-transfusion of RBCs collected on the second day. After storage at 2–8 °C overnight, plasma collected on the third day was separated from RBCs, preserved, and stored, while RBCs were discarded (i.e., they were not used for self-transfusion). After bleeding, horses were allowed to rest for 7 weeks before being re-immunized (Vaz and Araujo, 1949; Angulo et al., 1997b).

#### 2.3.3 Albumin-based fluid therapy

Following 7 weeks at rest after immunization and bleeding, two groups of three horses each were immunized and bled as described above. Then, immediately after the bleeding on the third day, the first group received an intravenous infusion of equine albumin at a dose of 2 g/kg body weight, in a total volume of 8–9 L. Equine albumin was purified by the method of aqueous two-phase system (Vargas et al., 2015) from plasma previously collected from the same group of horses. The solution was sterile, endotoxin-free and formulated at 10 g/dL total protein, with a purity of 70% (Supplementary Fig. S1). In addition to albumin, these preparations also contain other plasma proteins (mainly immunoglobulins and fibrinogen). The second group was used as a control with no albumin administration. After administration of the albumin-based fluid therapy, both groups were allowed to rest for seven weeks.

### 2.4 Cardiac electrophysiology

Heart rate, pulse oximetry, blood pressure, and electrocardiogram were determined by using a Cardell Max-1 model cardiac monitor (Midmark, Versailles, Ohio). To control oxygenation, a central line was established in the mandibular artery. To obtain the base-apex lead, the positive electrode was placed in the fifth intercostal space on the left side, at the level of the elbow; the neutral electrode on the back, at a point away from the heart; and the negative electrode on the skin of the right jugular groove, two-thirds of the way from the right mandibular ramus to the chest inlet. Periodically, the electrodes were sprayed with alcohol to facilitate the electrical transfer from the animal. Collection of data was carried out before the bleeding and throughout the process until the blood collection and the self-transfusions of RBCs suspensions were completed.

### 2.5 Serum biochemical and hematological analyses

Serum biochemical and hematological analyses were performed in samples of each individual horse. Blood samples were collected from horses after the rest period following the last bleeding (basal state), after immunization, after bleeding, after albumin-based fluid therapy, and during recovery. For the serum biochemical analyses, samples were collected from the jugular vein and left to clot at room temperature (20–25 °C); then, the sera were separated by centrifugation and processed in a clinical chemistry analyzer (Spin200 E Automatic biochemistry analyzer; Spinreact, Barcelona, Spain). Creatine kinase (CK) was determined by the corresponding International Federation of Clinical Chemistry and Laboratory Medicine (IFCC) method. Creatinine was quantified by a kinetic modification of the Jaffe colorimetric method (Mazzachi et al., 2000) and blood urea nitrogen (BUN) by a modification of the Talke and Schubert method (Talke and Schubert, 1965). Chloride ion concentration in the samples was determined by their reaction with mercury thiocyanate to produce a complex whose absorbance at 480 nm is proportional to the concentration of chloride ions in the sample. Sodium was quantified enzymatically, through the sodium-dependent β-galactosidase activity on o-nitrophenyl-β-D-galactopyranose to generate a product whose absorbance at 405 nm is proportional to the sodium concentration. Potassium was determined by a kinetic assay of the potassium-dependent pyruvate kinase which ends with the conversion of NADH to NAD and the corresponding decrease in absorbance at 380–405 nm, which is proportional to potassium concentration. The activity of aspartate transaminase (AST) was determined by the corresponding IFCC method. Gamma-glutamyl transferase (GGT) was determined by a modification of the Szasz procedure (Szasz, 1969). Albumin concentration was assessed by the bromocresol green colorimetric method (Rodkey, 1965). The concentration of globulins was calculated by subtracting the albumin concentration from the total protein concentration determined by the Biuret method (Gornall et al., 1949). For hematological analyses, the blood was collected from the jugular vein, anticoagulated with EDTA and then processed in a Veterinary Hematology Analyzer (Exigo Eos Hematology System; Boule Diagnostics AB, Stockholm, Sweden).

### 2.6 Determination of plasma volume

The plasma volume of the six horses used to study the effect of the albumin-based fluid therapy in the recovery after bleeding was determined before and 3 days after bleeding, and thereafter, once a week for 7 weeks. In each determination, a basal blood sample was collected in heparinized tubes. Horses then received an intravenous injection, in the jugular vein, of a solution of Evans Blue dye at a dose of 1 mg/kg body weight. One hour later, a second blood sample was collected from the contralateral jugular vein using EDTA as anticoagulant. Plasma from both samples was separated by centrifugation. The concentration of Evans Blue was determined by recording the Relative Fluorescence Units (RFUs) of the samples in a Cytation 3 Imaging Reader (Biotek), with excitation and emission wavelengths of 470 and 680 nm, respectively, and interpolating these values in a calibration curve constructed with standards of different concentrations of Evans Blue dye dissolved in normal equine serum. The concentrations of samples collected after the injection of Evans Blue were corrected by subtracting the concentrations in the corresponding basal samples from horses prior to the injection of Evans blue solution. Finally, the plasma volume was calculated as the ratio of the mass of Evans Blue that was injected to its corrected plasma concentration.

### 2.7 Statistical analyses

The statistical significance of the differences observed between basal values of serum biochemical and hematological parameters and those after immunization, bleeding and rest was evaluated by the Student's *t*-test in paired samples. The same analysis was used to evaluate the differences in plasma volume and biochemical parameters between horses receiving intravenous albumin and comparable animals used as controls. P values < 0.05 were considered significant.

## 3 Results and discussion

### 3.1 Basal condition of horses before immunization and bleeding

Primary immunization of horses used in this study was performed by the injection of venoms of *B. arietans*, *E. ocellatus*, *D. polylepis,* and *N. nigricollis*, following a procedure described by Gutiérrez et al. (2005). Since then and for several years (i.e., a range of 2–10 years), these horses have been regularly re-immunized and bled to produce hyperimmune plasma for the manufacture of the antivenom EchiTAb-ICP. In these re-immunization schemes, venoms are diluted in saline solution without adjuvants. The basal condition of all horses in this experiment corresponds to the seventh week of rest after the last immunization and bleeding cycles (Table 1, Table 2).

**Table 1:** Serum biochemical parameters of horses throughout immunization, bleeding and recovery.

Plasma biochemical parameter^a^	Basal	Ten days after immunization	After bleeding	
			3 days	7 weeks
CK (136–401 IU/L)	386 ± 206	442 ± 387	509 ± 295^b^	334 ± 204
Creatinine (11–164 μmol/L)	113 ± 16	100 ± 15^b^	93 ± 14^b^	91 ± 14^b^
BUN (5–12 mol/L)	11 ± 2	7 ± 1^b^	6 ± 1^b^	7 ± 1^b^
Na^+^ (133–141 mmol/L)	154 ± 12	143 ± 7^b^	144 ± 4^b^	103 ± 4^b^
Cl^−^(94–104 mmol/L)	98 ± 4	102 ± 2^b^	102 ± 2^b^	103 ± 2^b^
K^+^ (2.1–4.6 mmol/L)	4.5 ± 0.4	3.8 ± 0.3^b^	3.6 ± 0.3^b^	2.9 ± 0.4^b^
AST (150–464 IU/L)	321 ± 86	302 ± 69	287 ± 61^b^	400 ± 78^b^
GGT (15–49 IU/L)	33 ± 18	45 ± 27^b^	34 ± 19	36 ± 17
Albumin (2.7–3.7 g/dL)	3.6 ± 0.3	4.5 ± 0.4^b^	4.4 ± 0.3^b^	4.7 ± 0.2^b^
Globulins (3.0–4.9 g/dL)	5.2 ± 0.7	6.1 ± 0.8^b^	5.3 ± 0.9	5.0 ± 0.8
Albumin/globulin ratio	0.69	0.73	0.83	0.94
Total protein (6.3–8.0 g/dL)	8.8 ± 0.8	10.6 ± 0.9^b^	9.8 ± 0.7^b^	9.6 ± 0.7^b^

a Intervals of normal values for creole horses determined by the Laboratory of Chemical Analyses of the School of Veterinary Medicine, Universidad Nacional, Costa Rica, LAC-UNA are indicated in parentheses. CK: creatine kinase; BUN: Blood urea nitrogen; AST: Aspartate transaminase; GGT: Gamma-glutamyl transferase. Results are presented as mean ± SEM (n = 38).

b Significant differences when compared to basal values (P < 0.05).

**Table 2:** Hematological parameters of horses throughout immunization, bleeding and recovery.

Hematological parameter^a^	Basal	Ten days after immunization	After bleeding	
			3 days	7 weeks
RBC (7.5–10.5) x10^12^/L	9.0 ± 1.0	9.1 ± 0.9	6.8 ± 0.8^b^	8.2 ± 0.9^b^
HCT (38–50%)	42.0 ± 4.0	42.3 ± 3.7	31.3 ± 3.4^b^	39.0 ± 3.7^b^
HGB (13.3–17.9 g/dL)	16.1 ± 1.6	16.2 ± 1.5	12.1 ± 1.4^b^	15.0 ± 1.5^b^
MVC (43.2–52.8) fl	46.7 ± 2.4	46.3 ± 2.2^b^	46.1 ± 2.3^b^	47.5 ± 2.7^b^
MCH (15.0–18.5 pg)	17.9 ± 0.8	17.8 ± 0.8^b^	17.8 ± 0.8^b^	18.3 ± 1.0^b^
MCHC (33.7–39.0 g/dL)	38.4 ± 0.6	38.5 ± 0.6^b^	38.7 ± 0.7^b^	38.6 ± 0.6^b^
WBC (6.0–12.5 × 10^9^/L)	10.0 ± 1.7	10.8 ± 1.6^b^	9.5 ± 1.8^b^	10.7 ± 1.8^b^
GRAN (43–83%)	53.0 ± 8.2	57.1 ± 8.1^b^	54.4 ± 6.4	54.1 ± 7.3
LYM (10–48%)	38.4 ± 8.1	35.1 ± 7.7^b^	37.9 ± 6.0	37.8 ± 7.1
MONO (0–10%)	8.6 ± 1.0	7.8 ± 0.9^b^	7.7 ± 0.7^b^	8.1 ± 0.9^b^
Platelets (60–261) x10^9^/L	147 ± 58	157 ± 57^b^	146 ± 48	142 ± 51
MPV (3.9–6.5 ft)	4.7 ± 0.6	4.8 ± 0.6	4.7 ± 0.5	4.6 ± 0.4^b^

a Intervals of normal values for creole horses determined by the Quality Control Laboratory of Instituto Clodomiro Picado are indicated in parentheses. RBC: Red blood cell count; HTC: Hematocrit; HGB: Hemoglobin concentration; MVC: Mean corpuscular volume; MCH: Mean corpuscular hemoglobin; MCHC: Mean corpuscular hemoglobin concentration; WBC: White blood cell count; GRAN: Granulocytes; LYM: Lymphocytes, MONO: Monocytes; MPV: Mean platelet volume. Results are presented as mean ± SD (n = 38).

b Significant differences regarding basal values (P < 0.05).

At the basal condition, the values of CK, AST, GGT, creatinine, blood urea nitrogen, and albumin were within the reference ranges for horses (Table 1), thus indicating that no muscle, heart, hepatic, and renal damage occurred in these horses prior to the re-immunization process. A mild hypernatremia was detected (Table 1). As the horses were maintained with water *ad libitum* and they have normal serum values of chloride and potassium, this finding is likely due to excessive sodium intake, which could be easily corrected by diet adjustments.

As is usual in hyperimmunized animals, there was a higher concentration of globulins and total proteins in serum, as compared to reference ranges, which is the consequence of active immunoglobulin synthesis (Table 1; Estrada et al., 1992; Angulo et al., 1997a). Otherwise, before re-immunization, all the horses showed a normal hematologic profile, without evidence of anemia or dehydration (Table 2).

### 3.2 Effect of re-immunization with venoms

After venom injection, venom components such as metalloproteinases (SVMPs), phospholipases A_2_ (PLA_2_s), and three-finger toxins (3FTxs), among others, exert their toxic effects in the tissues surrounding the injection site.

After 10 days, a 10–15 cm diameter area of mild swelling developed at the injection site in some horses, but none of the animals immunized developed abscesses, fistulas, or fibrosis. This contrasts with the results obtained by immunizing horses with these venoms but mixed with various types of emulsion adjuvants (Arguedas et al., 2022) or with viperid venoms from Costa Rica (Angulo et al., 1997). This discrepancy is explained by the fact that in the present study, immunogens were formulated without adjuvants, which are largely responsible for the development of local lesions (Arguedas et al., 2022). In agreement with macroscopic observations, no significant increment in the serum concentration of 10.13039/501100001515CK, as compared to the basal value, was observed (*t* = −0.869; df = 37; P = 0.391; Table 1), hence supporting the lack of local myotoxicity.

Since snake venoms have the potential to induce systemic toxicity through the direct effect of toxins on tissues, and the indirect effects secondary to systemic inflammation, it was of interest to assess several laboratory parameters associated with systemic alterations. Significant differences were observed between basal samples and those collected 10 days after immunization, regarding the serum concentrations of creatinine (*t* = 6.224; df = 37; P = 0.000; Table 1), urea nitrogen (*t* = 15.695; df = 37; P = 0.000; Table 1), chloride (*t* = −7.368; df = 37; P = 0.000; Table 1), potassium (*t* = 10.233; df = 37; P = 0.000; Table 1) and GGT (*t* = −3.714; df = 37; P = 0.001; Table 1). However, most of these parameters remained within the reference range (Table 1). Moreover, in most of these cases, the values in samples 10 days after venom injection were significantly lower than those of basal samples before re-immunization (Table 1). Taken together, these observations suggest that horses submitted to the re-immunization process hereby described did not develop significant local and systemic tissue damage as a consequence of venom injection.

As expected, the injection of venom stimulated the synthesis of antivenom antibodies (i.e., gamma globulins), which are responsible for most of the significant rise in serum globulins (*t* = −8.282; df = 37; P = 0.000; Table 1) and total protein concentration (*t* = −12.252; df = 37; P = 0.000; Table 1) 10 days after immunization. These antibodies conferred the plasma with the ability to neutralize the homologous venoms (i.e., the venoms used during the immunization) and cross-neutralize other antigenically related venoms (Segura et al., 2010; Gómez et al., 2022).

Regarding the changes observed in several hematological parameters in re-immunized horses, even though some of them showed significant differences when compared to basal values, all of them were within the reference intervals for horses (Table 2). These observations suggest that re-immunization with venoms, at the doses used in this study, did not induce anemia, leukocytosis, nor thrombocytopenia.

### 3.3 Effect of bleeding

To assess the effect of bleeding on biochemical and hematological parameters, values of samples collected three days after bleeding were compared to basal values of horses before re-immunization and with the horse reference interval. The comparison of the basal samples with those collected three days after bleeding showed that significant effects on some parameters of the serum biochemistry of horses were induced as a consequence of loss of blood (Table 1). However, in most cases, the values were within the reference ranges established for horses. The only two parameters that deviated from the reference range were the CK activity and the sodium concentration in serum. In the case of CK, this might reflect a somehow delayed damage to skeletal muscle as a consequence of venom injection. In the case of sodium, there was a drop in serum levels as compared to basal values, approaching the reference range (Table 1). Regarding serum proteins, there was a significant increment in the concentrations of albumin and total protein as compared to basal values (Table 1).

We were interested in analyzing the previous biochemical findings in light of the overall plasma loss during the bleeding protocol. The blood volume in horses is close to 10% of their body weight (i.e., 400–450 kg), meaning that each animal has between 40 and 45 L of blood. Considering the mean basal hematocrit (i.e., 42%), the volume of plasma per horse corresponds to 23–26 L. The mean basal albumin concentration before bleeding was 4.5 ± 0.3 g/dL, implying that the total amount of plasma albumin was approximately 1044–1174 g per horse. On the other hand, the 8 L of plasma collected from each animal at the end of the third bleeding day entails that approximately 360 g of albumin was lost per horse. Consequently, the total amount of plasma albumin in the horses was reduced during bleeding to 684–814 g per animal.

Assuming that plasma volume recovers faster than plasma albumin, due to the redistribution of fluid between the interstitial tissue compartment and the vascular compartment, the concentration of serum albumin three days after bleeding should be close to 2.9–3.1 g/dL. However, the experimental values (i.e., 4.4 ± 0.3 g/dL; Table 1) were higher than these theoretical predictions. This finding, similar to what was obtained by Feige et al. (2005), could be due to slow rehydration and/or a fast albumin replacement. More likely, it could be explained by the action of homeostatic mechanisms aimed to regulate the serum concentration of albumin, more likely by the mobilization from the extravascular compartment, where most of the total body albumin is contained (Merlot et al., 2014).

When hematological parameters were analyzed and compared to control values, significant reductions were observed in values three days after bleeding: RBC (t = 16.121; df = 37; P = 0.000; Table 2), hematocrit (t = 16.649; df = 37; P = 0.000; Table 2), and hemoglobin (t = 16.433; df = 37; P = 0.000; Table 2), as well as in the parameters calculated from these values (Table 2). Of these, only RBC, hematocrit and hemoglobin were outside the range of reference values. These reductions are the consequence of the net loss of RBCs during the bleeding protocol implemented since no RBC reinfusion was done after the third day. Similar findings were described in horses submitted to bleeding during the manufacture of the polyvalent viperid antivenom used in Costa Rica (Angulo et al., 1997b).

Despite the loss of blood associated with the bleeding protocol, no relevant variations of oxygen saturation (SpO_2_ ≥ 90%), systolic pressure (140–190 mm Hg), diastolic pressure (90–110 mm Hg), heart rate (27–43 bpm), P wave duration (0.09–0.14 s), PR interval (0.21–0.33 s), or QRS duration (0.12–0.14 s) were detected during the bleeding of three horses studied for cardiorespiratory alterations (Welch-Huston et al., 2020). The loss of RBCs during bleeding is likely to be compensated by splenic contraction and the consequent mobilization of cells into the intravascular compartment (Udroiu, 2017). Further research is needed to understand the mechanisms involved in the recovery of hematological parameters after the bleeding protocols are carried out in antivenom production.

Despite the large volume of blood removed from horses in the followed bleeding protocol, our results suggest that horses have efficient mechanisms to assimilate and endure the loss of blood without suffering major biochemical, cardiorespiratory, or hematological alterations (Table 1, Table 2; Schmotzer et al., 1985; Malikides et al., 2001).

### 3.4 Recovery time after bleeding and the effect of albumin-based fluid therapy

When biochemical and hematological parameters were analyzed seven weeks after the bleeding protocol, several parameters showed a significant difference compared to basal values (Table 1, Table 2). However, the majority of parameters were within the reference normal ranges of horses, thus highlighting the recovery at this time interval after bleeding. The only exception was the concentration of serum proteins, which was above the upper limit of the reference range (Table 1, Table 2). This is likely to depend on the increased protein synthesis associated with the immune response to venom injection.

Our findings agree with previous studies reporting that after 8 L of blood are drawn, horses require approximately one to four weeks to replenish their RBC count (Magdesian et al., 1992; Angulo et al., 1997b), about 3–4 weeks to recover their total plasma protein concentration (Magdesian et al., 1992), and 4–7 weeks to fully replenish their extravascular albumin stores (Fleck and Colley, 1984; Magdesian et al., 1992). Overall, our observations indicate that horses have set of biochemical and hematological parameters within normal range seven weeks after bleeding.

**Fig. 1:**
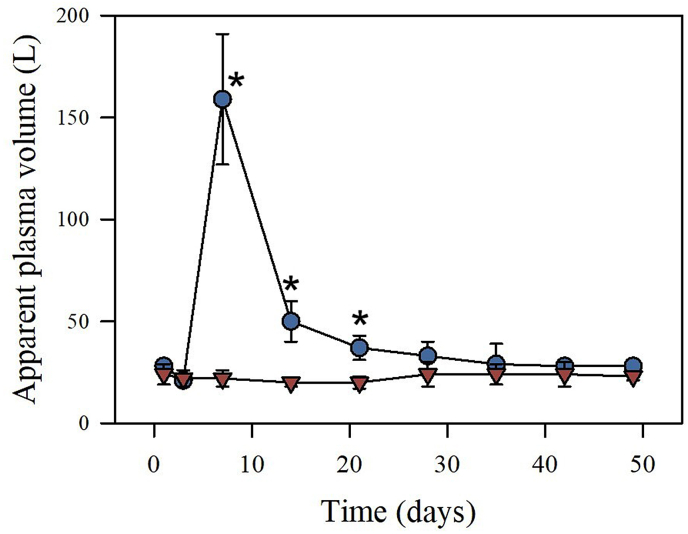
Effect of albumin-based therapy in the apparent plasma volume of horses after immunization with snake venoms and bleeding. Blue circles and red triangles correspond to the groups receiving albumin and the control group, respectively. Values correspond to the mean ± SD (n = 3). *P < 0.05 when compared to the control group.

**Fig. 2:**
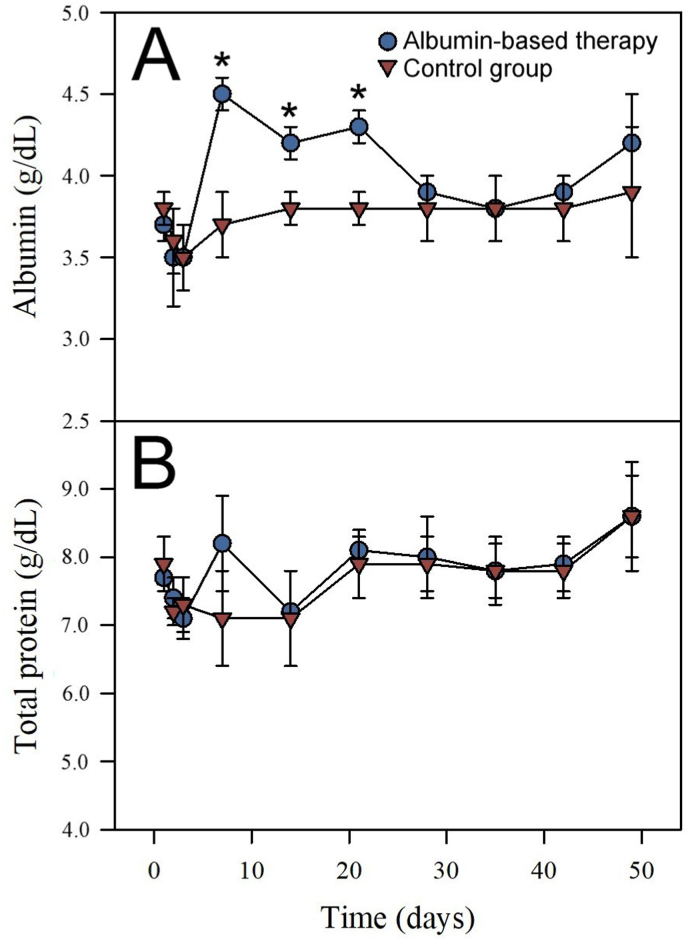
Effect of albumin-based therapy in the concentration of albumin (A) and total protein (B) of horses after immunization with snake venoms and bleeding. Blue circles and red triangles correspond to the group receiving albumin and the control group, respectively. Values correspond to the mean ± SD (n = 3). *P < 0.05 when compared to the control group.

In order to assess whether the administration of horse albumin would speed up the recovery of horses before the next bleeding session, the effect of the albumin-based fluid therapy in the time required to normalize the plasma volume and recover the total protein lost during bleeding was tested in two groups of three horses (i.e., the test and control groups). The mean basal plasma volume was 27.7 ± 3.2 and 24.0 ± 4.6 L for the test and control groups, respectively (Fig. 1). The mean basal concentration of albumin was 3.7 ± 0.1 and 3.8 ± 0.1 g/dL for the test and control groups, respectively (Fig. 2A). The mean basal concentration of total protein was 7.7 ± 0.1 and 7.9 ± 0.4 g/dL for the test and control groups, respectively (Fig. 2B). Moreover, the volume of plasma collected during the bleeding was 8.1 ± 0.4 L/horse. No significant differences in these parameters were observed when comparing the two experimental groups before the infusion of albumin.

Using the Evans Blue technique, the plasma volume of the six horses was estimated, obtaining 25.8 ± 4.4 L before bleeding and 21.3 ± 3.3 L after the third day of bleeding. The difference between these calculated volumes was not significant (t = 1.989; gl = 5; P = 0.103). Despite the imprecision inherent to this technique, the result underscores the activation of homeostatic mechanisms aimed at regulating plasma volume after the bleeding process.

Based on the bleeding protocol, we estimated that from the 624 g of total protein removed during the bleeding, around 300 g corresponded to albumin. So, once the bleeding was finished, the horses in the test group received a bolus of intravenous equine albumin at a dose of 2 g/kg body weight, whereas the control group did not receive any infusion. Within the first hour of albumin administration, all three horses developed a generalized rash, deep and labored breathing, and gastrointestinal disturbances (gastrointestinal sounds and flatulence), even though the albumin preparation administered passed the quality control tests (i.e., purity, sterility, and absence of endotoxins). These reactions were not observed in any horse of the control group. To manage albumin-induced adverse reactions, horses were treated with chlorpheniramine maleate (i.e., Histaminex) at a dose of 0.25 mg/kg and dexamethasone at a dose of 0.1 mg/kg. Twelve hours after treatment, none of the horses showed signs of adverse reactions.

Albumin administration resulted in a high and rapid increment of the apparent plasma volume, which was significantly higher when compared to the control group (Fig. 1). However, such increment (approximately 150 L) is much higher than the theoretical volume estimated based on the volume of albumin solution injected, hence evidencing an overestimation of the volume of plasma. This high plasma volume estimate could be explained through the binding of Evans blue to the albumin administered and possible extravasation of this protein, hence reducing the concentration of the dye in plasma. Then, calculations resulted in plasma volumes evidently greater than the real ones. Therefore, we use the term “apparent plasma volume” to refer to this observation.

**Fig. 3:**
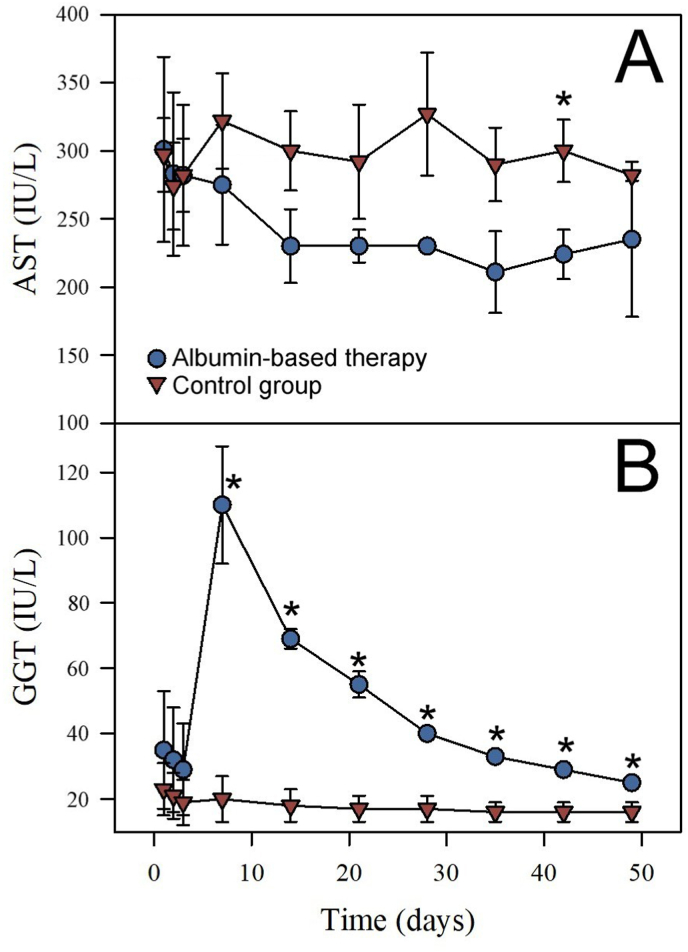
Effect of albumin-based therapy in the serum activity of AST (A) and GGT (B) of horses after immunization with snake venoms and bleeding. Blue circles and red triangles correspond to the group receiving albumin and the control group, respectively. Values correspond to the mean ± SD (n = 3). *P < 0.05 when compared to the control group.

Albumin administration resulted in a significant increment in the serum concentration of albumin (Fig. 2A) that was not reflected in the concentration of total protein (Fig. 2B). No significant differences in CK, creatinine, urea nitrogen, electrolytes, or hematologic parameters were detected between the two groups (not shown). Except for the samples at 42 days after albumin administration, no significant differences were observed in the serum concentration of AST between the test and control groups (Fig. 3A). On the other hand, after albumin administration, the test group showed values of GGT significantly higher than those corresponding to the control group (Fig. 3B). This result raises the possibility that the administration of albumin, as used here, may result in cholestasis or some hepatobiliary processes (Stockham, 1995; Mann et al., 2022). Throughout the rest period, plasma volume, albumin concentration, and GGT gradually diminished until they reached normal reference values. The intravenous administration of albumin did not hasten the recovery of these parameters in the horses.

## 4 Conclusions

Our findings indicate that the immunization and bleeding protocols applied to the horses used to produce the African polyspecific antivenom EchiTAb-plus-ICP do not induce overt alterations in the clinical, hematological and clinical chemistry parameters of horses that could compromise the health condition of these animals. After seven weeks of rest following immunization and bleeding, according to the parameters evaluated in this work, the horses recovered and showed laboratory parameters that were within the reference ranges described for horses. After the resting period, they were ready for the following immunization/bleeding cycle. Nevertheless, the search for the improvement of animal welfare during the production of antivenoms is always an unfinished work and further studies should be carried out to assess the effects of this and other immunization and bleeding protocols. The albumin-based fluid therapy used did not hasten the recovery of the horses; on the contrary, this procedure induced adverse effects and is, therefore, not recommended in the conditions described in this study.

## Credit author statement

Conceptualization: GL, JMG; Formal analysis: GL, JMG, AG; Funding acquisition: GL, JMG; Investigation: RH, MA, JME, EM, DU, GS, LB; Methodology: RH, MA, JME, LB, MVa, ÁS, AS, MH, MVi, GL; Project administration: GL; Resources: RH, MA, JME, LB, MVa, ÁS, AS, MH, MVi, GL; Writing-original draft preparation: GL, JMG, RH; Writing review and editing: RH, MA, JME, EM, EU, GS, LB, MVa, ÁS, AS, MH, MVi.

## Conflict of interest statement

Mauricio Arguedas, Edwin Moscoso, Deibid Umaña, Gabriela Solano, Mariángela Vargas, Alvaro Segura, Andrés Sánchez, María Herrera, Mauren Villalta, José María Gutiérrez, and Guillermo León work at Instituto Clodomiro Picado, where the antivenom EchiTAb-plus-ICP is manufactured. None of the authors of this paper has a financial or personal relationship with other people or organizations that could inappropriately influence or bias the content of the paper. Sponsors were not involved in the study design; in the collection, analysis, and interpretation of data; in the writing of the manuscript; or in the decision to submit the manuscript for publication.

## Ethical statement

This manuscript presents an experimental study performed following the standard procedure of scientific ethics, including the use and care of experimental animals. All procedures used in this study were approved by the Institutional Committee for the Care and Use of Laboratory Animals (CICUA) of Universidad de Costa Rica (Proceedings 82–08 and 39–20) and meet the International Guiding Principles for Biomedical Research Involving Animals (CIOMS, 1985).

## Declaration of competing interests

The authors declare the following financial interests/personal relationships which may be considered as potential competing interests:

Guillermo Leon Montero reports financial support was provided by Wellcome Trust.

## Data Availability

Data will be made available on request.

## Appendix A Supplementary data

figs1 figs1

## References

[bib1] Angulo Y., Estrada R., Gutiérrez J.M. (1997). Clinical and laboratory alterations in horses during immunization with snake venoms for the production of polyvalent (Crotalinae) antivenom. Toxicon.

[bib2] Angulo Y., Estrada R., Gutiérrez J.M. (1997). Effects of bleeding in horses immunized with snake venoms for antivenom production. Rev. Biol. Trop..

[bib3] Arguedas M., Umaña D., Moscoso E., García A., Pereira C., Sánchez A., Durán G., Cordero D., Sánchez A., Segura Á., Vargas M., Herrera M., Villalta M., Gómez A., Salas C., Díaz C., Gutiérrez J.M., León G. (2022). Comparison of adjuvant emulsions for their safety and ability to enhance the antibody response in horses immunized with African snake venoms. Vaccine X.

[bib4] Chotwiwatthanakun C., Pratanaphon R., Akesowan S., Sriprapat S., Ratanabanangkoon K. (2011). Production of potent polyvalent antivenom against three elapid venoms using a low dose, low volume, multi-site immunization protocol. Toxicon.

[bib5] CIOMS (Council of International Organizations of Medical Sciences) (1985). The international guiding Principles for biomedical research involving animals. Altern. Lab. Anim..

[bib6] da Silva W.D., Tambourgi D.V. (2011). The humoral immune response induced by snake venom toxins. Inflamm. Allergy - Drug Targets.

[bib7] Estrada R., Chaves F., Robles A., Rojas E., Segura E., Gutiérrez J.M. (1992). Hematologic values and serum enzymes in horses inoculated with snake venoms for the production of antivenins in Costa Rica. Rev. Biol. Trop..

[bib8] Feige K., Ehrat F.B., Kästner S.B., Wampfler B. (2005). The effects of automated plasmapheresis on clinical, haematological, biochemical and coagulation variables in horses. Vet. J..

[bib9] Fleck A., Colley C.M. (1984). How much plasma, relative to body weight, can a donor give over certain period without a continuous deviation of his plasma protein metabolism in the direction of plasma protein deficiency?. Vox Sang..

[bib10] Fukanoki S., Iwakura T., Iwaki S., Matsumoto K., Takeda R., Ikeda K., Shi Z., Mori H. (2001). Safety and efficacy of water-in-oil-in-water emulsion vaccines containing Newcastle disease virus haemagglutinin-neuraminidase glycoprotein. Avian Pathol..

[bib11] Gómez A., Sánchez A., Durán G., Cordero D., Segura Á., Vargas M., Solano D., Herrera M., Chaves-Araya S., Villalta M., Sánchez M., Arguedas M., Díaz C., Gutiérrez J.M., León G. (2022). Intrageneric cross-reactivity of monospecific rabbit antisera against venoms of the medically most important Bitis spp. and Echis spp. African snakes. PLoS Neglected Trop. Dis..

[bib12] Gornall A.G., Bardawill C.J., David M.M. (1949). Determination of serum proteins by means of the biuret reaction. J. Biol. Chem..

[bib13] Gutiérrez J.M., Rojas E., Quesada L., León G., Núñez J., Laing G.D., Sasa M., Renjifo J.M., Nasidi A., Warrell D.A., Theakston R.D., Rojas G. (2005). Pan-African polyspecific antivenom produced by caprylic acid purification of horse IgG: an alternative to the antivenom crisis in Africa. Trans. R. Soc. Trop. Med. Hyg..

[bib14] Jansen T., Hofmans M.P., Theelen M.J., Manders F.G., Schijns V.E. (2007). Dose and timing requirements for immunogenicity of viral poultry vaccine antigen: investigations of emulsion-based depot function. Avian Pathol..

[bib15] León G., Estrada R., Chaves F., Rojas G., Ovadia M., Gutiérrez J.M. (1999). Inhibition by CaNa2EDTA of local tissue damage induced by Bothrops asper (terciopelo) venom: application in horse immunization for antivenom production. Toxicon.

[bib16] León G., Sánchez L., Hernández A., Villalta M., Herrera M., Segura A., Estrada R., Gutiérrez J.M. (2011). Immune response towards snake venoms. Inflamm. Allergy - Drug Targets.

[bib17] León G., Vargas M., Segura Á., Herrera M., Villalta M., Sánchez A., Solano G., Gómez A., Sánchez M., Estrada R., Gutiérrez J.M. (2018). Current technology for the industrial manufacture of snake antivenoms. Toxicon.

[bib18] Magdesian K.G., Brook D., Wickler S.J. (1992). Temporal effects of plasmapheresis on serum proteins in horses. Am. J. Vet. Res..

[bib19] Malikides N., Hodgson J.L., Rose R.J., Hodgson D.R. (2001). Cardiovascular, haematological and biochemical responses after large volume blood collection in horses. Vet. J..

[bib20] Mann S., Ramsay J.D., Wakshlag J.J., Stokol T., Reed S., Divers T.J. (2022). Investigating the pathogenesis of high-serum gamma-glutamyl transferase activity in Thoroughbred racehorses: a series of case-control studies. Equine Vet. J..

[bib21] Mazzachi B.C., Peake M.J., Ehrhardt V. (2000). Reference range and method comparison studies for enzymatic and Jaffé creatinine assays in plasma and serum and early morning urine. Clin. Lab..

[bib22] Merlot A.M., Kalinowski D.S., Richardson D.R. (2014). Unraveling the mysteries of serum albumin-more than just a serum protein. Front. Physiol..

[bib23] Rodkey F.L. (1965). Direct spectrophotometric determination of albumin in human serum. Clin. Chem..

[bib24] Schmotzer W.B., Riebold T.W., Porter S.L., Blauvelt S.R. (1985). Time saving techniques for the collection, storage, and administration of equine blood and plasma. Vet. Med..

[bib25] Segura A., Villalta M., Herrera M., León G., Harrison R., Durfa N., Nasidi A., Calvete J.J., Theakston R.D., Warrell D.A., Gutiérrez J.M. (2010). Preclinical assessment of the efficacy of a new antivenom (EchiTAb-Plus-ICP) for the treatment of viper envenoming in sub-Saharan Africa. Toxicon.

[bib26] Stockham S.L. (1995). Interpretation of equine serum biochemical profile results. Vet. Clin. N. Am. Equine Pract..

[bib27] Szasz G. (1969). A kinetic photometric method for serum gamma-glutamyl transpeptidase. Clin. Chem..

[bib28] Talke H., Schubert G.E. (1965). Enzymatic urea determination in the blood and serum in the Warburg optical test. Klin. Wochenschr..

[bib29] Theakston R.D., Warrell D.A., Griffiths E. (2003). Report of a WHO workshop on the standardization and control of antivenoms. Toxicon.

[bib30] Udroiu I. (2017). Storage of blood in the mammalian spleen: an evolutionary perspective. J. Mamm. Evol..

[bib31] Vargas M., Segura Á., Villalta M., Herrera M., Gutiérrez J.M., León G. (2015). Purification of equine whole IgG snake antivenom by using an aqueous two phase system as a primary purification step. Biologicals.

[bib32] Vaz E., Araujo P. (1949). Da sangria de animais de imunização. Mem. Inst. Butantan (Sao Paulo).

[bib33] Waghmare A.B., Salvi N.C., Deopurkar R.L., Shenoy P.A., Sonpetkar J.M. (2014). Evaluation of health status of horses immunized with snake venom and montanide adjuvants, IMS 3012 (nanoparticle), ISA 206 and ISA 35 (emulsion based) during polyvalent snake antivenom production: hematological and biochemical assessment. Toxicon.

[bib34] Welch-Huston B., Durward-Akhurst S., Norton E., Ellingson L., Rendahl A., McCue M. (2020). Comparison between smartphone electrocardiography and standard three-lead base apex electrocardiography in healthy horses. Vet. Rec..

[bib35] WHO (World Health Organization) (2016).

