# Supplementary figures and images for: Clinical effects of immunization, bleeding, and albumin-based fluid therapy in horses used as immunoglobulin source to produce a polyspecific antivenom (Echitab-plus-ICP) towards venoms of African snakes
Source: Toxicon X. 2023 Apr 17;18:100158. doi: 10.1016/j.toxcx.2023.100158 (PMC10172988; doi:10.1016/j.toxcx.2023.100158)

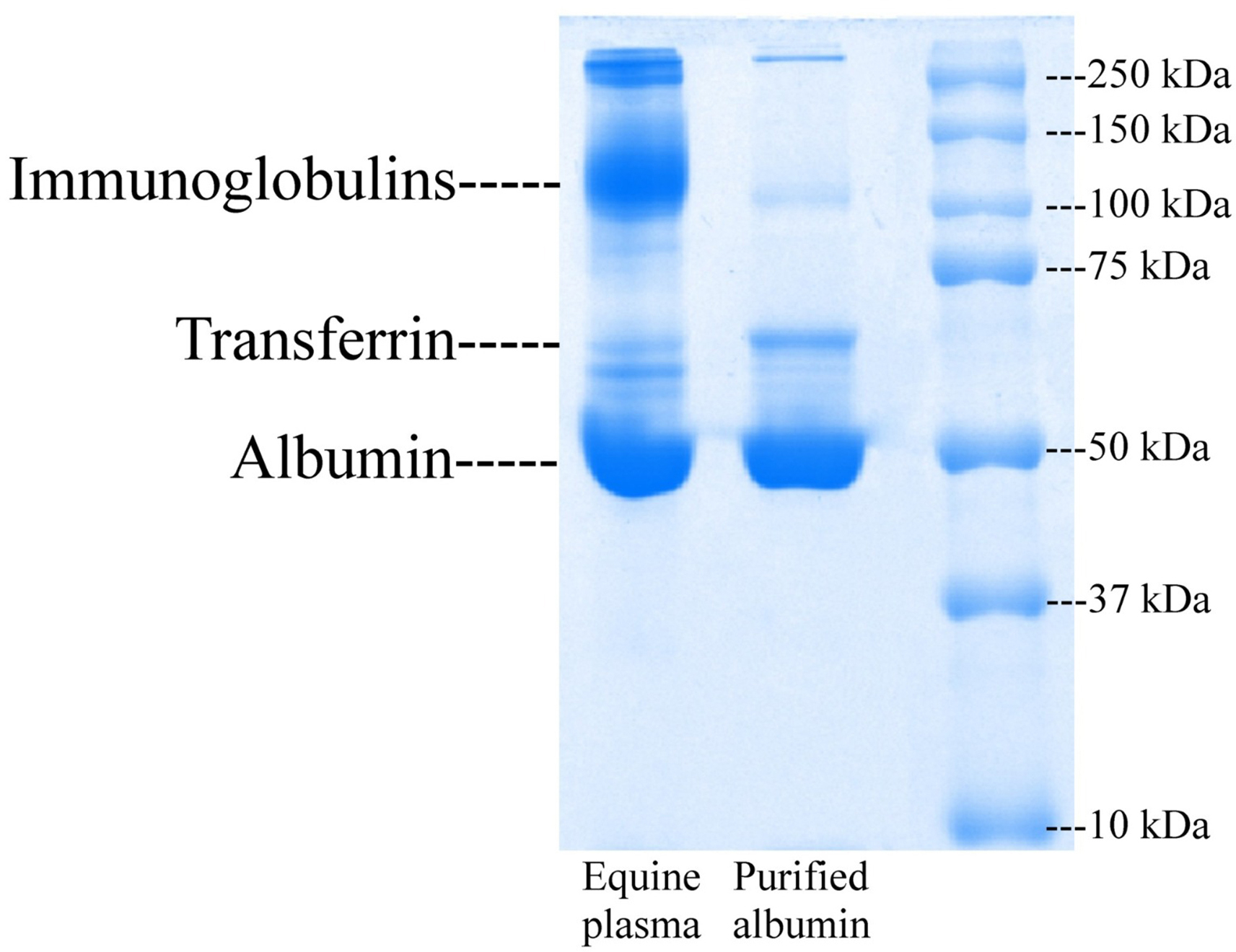

Supplement: figs1 [file mmcfigs1.jpg]
